# Soil, Plant, and Microorganism Interactions Drive Secondary Succession in Alpine Grassland Restoration

**DOI:** 10.3390/plants13060780

**Published:** 2024-03-09

**Authors:** Chenglong Han, Defei Liang, Weidi Zhou, Qiuyun Xu, Mingxue Xiang, Yanjie Gu, Kadambot H. M. Siddique

**Affiliations:** 1 State Key Laboratory of Plateau Ecology and Agriculture, Qinghai University, Xining 810016, China; hanchenglong2008@126.com (C.H.); liang-df221@qhu.edu.cn (D.L.); 2023990034@qhu.edu.cn (M.X.); 2 College of Agriculture and Animal Husbandry, Qinghai University, Xining 810016, China; zhouweidi010206@163.com (W.Z.); xuqiuyun1996@163.com (Q.X.); 3 The UWA Institute of Agriculture, The University of Western Australia, Perth, WA 6001, Australia; kadambot.siddique@uwa.edu.au

**Keywords:** recovery of grassland, vegetation types, soil nutrient cycles, microbial community composition, succession

## Abstract

Plant secondary succession has been explored extensively in restoring degraded grasslands in semiarid or dry environments. However, the dynamics of soil microbial communities and their interactions with plant succession following restoration efforts remain understudied, particularly in alpine ecosystems. This study investigates the interplay between soil properties, plant communities, and microbial populations across a chronosequence of grassland restoration on the Qinghai–Tibet Plateau in China. We examined five succession stages representing artificial grasslands of varying recovery durations from 0 to 19. We characterized soil microbial compositions using high-throughput sequencing, enzymatic activity assessments, and biomass analyses. Our findings reveal distinct plant and microbial secondary succession patterns, marked by increased soil organic carbon, total phosphorus, and NH_4_^+^-N contents. Soil microbial biomass, enzymatic activities, and microbial community diversity increased as recovery time progressed, attributed to increased plant aboveground biomass, cover, and diversity. The observed patterns in biomass and diversity dynamics of plant, bacterial, and fungal communities suggest parallel plant and fungal succession occurrences. Indicators of bacterial and fungal communities, including biomass, enzymatic activities, and community composition, exhibited sensitivity to variations in plant biomass and diversity. Fungal succession, in particular, exhibited susceptibility to changes in the soil C: N ratio. Our results underscore the significant roles of plant biomass, cover, and diversity in shaping microbial community composition attributed to vegetation-induced alterations in soil nutrients and soil microclimates. This study contributes valuable insights into the intricate relationships driving secondary succession in alpine grassland restoration.

## 1. Introduction

The Qinghai–Tibetan region in western China, encompassing an estimated 3.58 million ha of swampy meadows [1], faces widespread degradation due to climate change and insufficient protection awareness. Intensity livestock grazing and exploitation have produced *Heitutan* landscapes with extremely sparse vegetative cover or complete denudation [1,2]. Efforts to restore degraded swampy meadows have become a significant area of research, aiming to understand causative factors and develop effective control measures [3,4,5]. Establishing artificial grasslands is a promising and efficient approach for restoring severely degraded alpine meadows [6,7], but our understanding of these processes in alpine environments remains limited.

Soil microorganisms are crucial indicators of soil health [6] and game-changers in restoring degraded soil functions [7]. Their impact on plant growth, through organic matter decomposition and nutrient availability maintenance, extends to impacting plant biomass, richness, and diversity [8,9]. In turn, changes in plant litter, roots, and root exudation regulate soil microbial community composition, activity, and diversity during ecosystem restoration [10,11]. The reciprocal relationship between changes in plant community properties and microbial community composition influences plant community and soil microorganism dynamics [12,13]. While previous studies on degraded grassland restoration have focused on plant communities, soil nutrients, microbial activity, and community composition [14,15], the interplay between plants and soil microbial communities during vegetation secondary succession, especially in alpine regions, remains underexplored.

Soil microbes, particularly bacteria and fungi, are the dominant decomposers of plant litter. Their activity—which significantly impacts biogeochemical processes and plant community establishment—is highly influenced by environmental conditions [16]. Moreover, their patterns of secondary succession during recovery and response to soil nutrient levels vary due to differences in life history [17]. Fungi, known for decomposing recalcitrant substrates and mediating slow carbon (C) cycling pathways in soil [18], exhibit distinct succession patterns compared to bacteria, which generally regulate rapid soil biochemical cycling with faster growth rates [19]. However, microbial growth limitations due to variations in nutrient stoichiometry during plant succession can influence the accumulation of carbon sources from litter and roots and their exudation [10,11,20,21]. Understanding how soil microbial communities respond to plant succession and soil nutrient changes is crucial for comprehending the impacts of alpine grassland restoration on soil health.

In this study, we selected five grasslands with varying recovery periods (0, 5, 14, 16, and 19 years) in a typical alpine area on the Qinghai–Tibetan Plateau of China to investigate temporal changes in soil properties, plant communities, and microbial community structures during the long-term chronosequence of recovery. Our specific objectives were to (1) characterize changes in plant productivity (biomass), soil properties (nutrient levels), and soil microbial communities (activity, richness, and diversity) at different vegetation recovery times, (2) assess the consistency of plant succession patterns with those of soil microbial communities, and (3) explore the relationships among soil properties, plant communities, and microbial populations along the chronosequence. In the unique alpine environment, we anticipated uncovering the relationship between soil microbial community succession and aboveground plant changes following artificial grassland restoration and identifying key environmental factors driving succession.

## 2. Results

### 2.1. Plant Communities

Plant community properties significantly differed with increasing recovery time. Recovery time (R5, R14, R16, and R19) significantly increased plant aboveground biomass, coverage, richness, and diversity (*p <* 0.05) compared to R0 (Table 1). The Shannon index and biomass followed a similar increasing pattern, peaking at R14. Plant coverage significantly increased at R14 and R19 compared to R0. Perennial species richness was notably higher at R19 than at R0.

### 2.2. Soil Properties and Microbial Activity

Recovery time significantly influenced soil nutrients, microbial biomass, and enzyme activities (Table 2). Plant recovery time significantly (*p* < 0.05) increased soil organic carbon (SOC) content, with similar trends for total nitrogen (TN), total phosphorus (TP), available phosphorous (AP), and ammonium nitrogen (NH_4_^+^-N) contents. Conversely, nitrate nitrogen (NO_3_N) content gradually decreased during the grassland recovery, reaching its lowest point at R19. The C: N ratio initially decreased in the first five years of recovery compared to R0 but then increased over time. Interestingly, soil microbial biomass carbon and nitrogen (MBC and MBN) generally decreased with increasing recovery time. Grassland recovery significantly enhanced β-1,4-glucosidase (BG), β-1,4-N-acetylglucosaminidase (NAG), cellobiohydrolase CBH, acid phosphatase (ACP), and phenoloxidase (POX) activities, relative to R0.

### 2.3. Soil Microbial Community Composition

A total of 13,319 operational taxonomic units (OTUs) with 97% similarity were identified, with no significant (*p* < 0.05) difference in the number of OTUs between R0 and R19 (Table 3). The bacteria community richness evaluation, using Chao1 and abundance-based coverage estimator (ACE), revealed a significant (*p* < 0.05) decrease during the first 14 years of grassland recovery relative to R0, followed by an increase over time. In contrast, Chao1 and ACE estimators of fungal community richness generally decreased with recovery time. The Shannon index in the bacterial community, reflecting diversity, exhibited a similar pattern to Chao1 and ACE, with the highest values at R19. However, the Shannon index for fungi generally decreased with recovery time, reaching its lowest value at R19.

**Figure 1:**
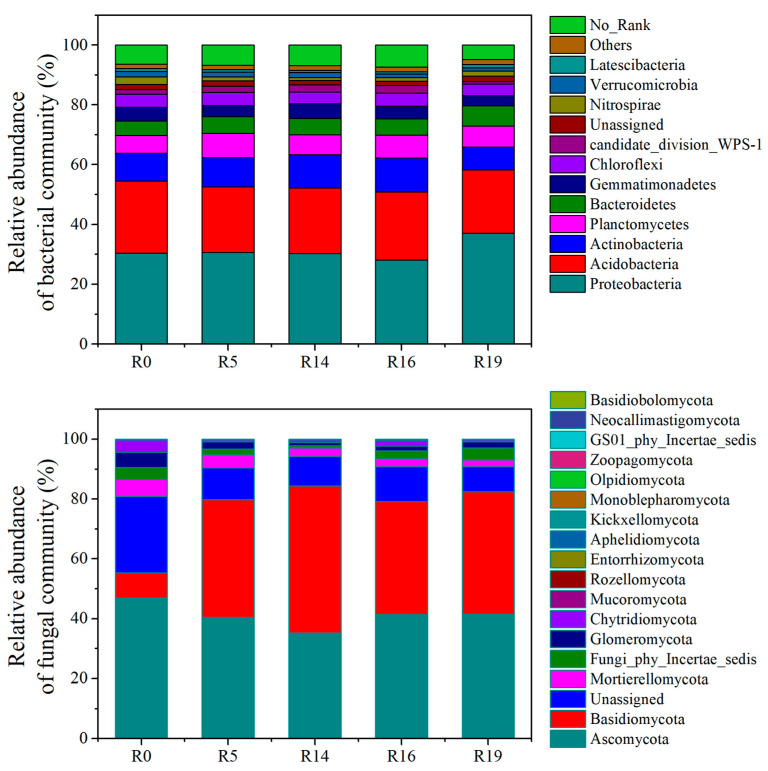
Relative abundance of soil bacterial and fungal communities with secondary succession time at the phylum level. R0: highly degraded grassland; R5: recovery time of 5 years; R14: recovery time of 14 years; R16: recovery time of 16 years; R19: recovery time of 19 years.

**Figure 2:**
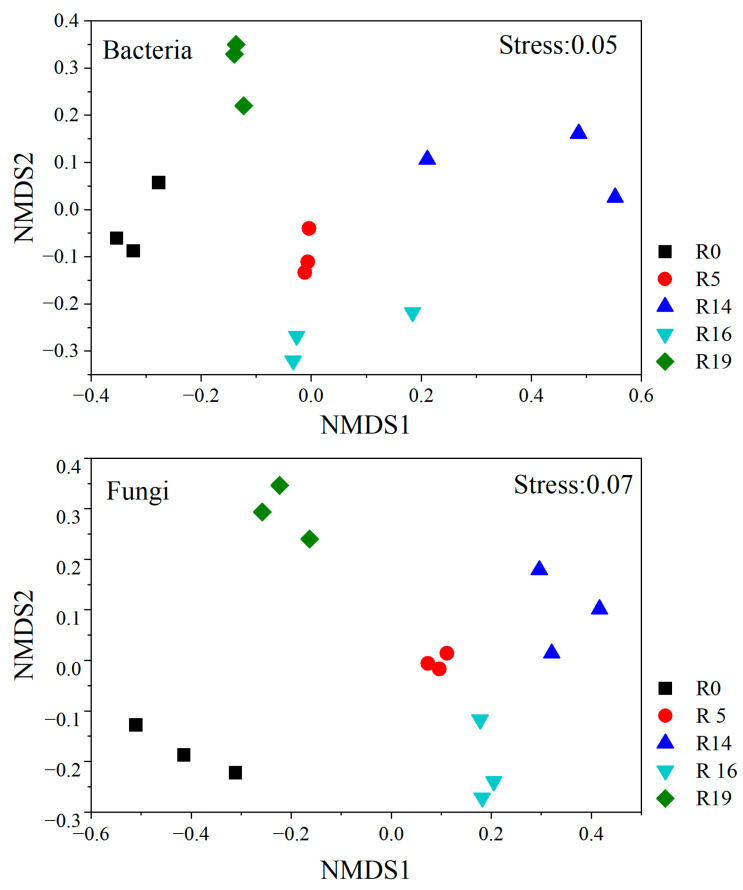
Non-metric multi-dimensional scaling analysis of bacterial and fungal communities (OTU abundance) under different treatments (R0, R5, R14, R16, and R19). R0: highly degraded grassland; R5: recovery time of 5 years; R14: recovery time of 14 years; R16: recovery time of 16 years; R19: recovery time of 19 years.

The identified OTUs were categorized into ≥17 fungal and 11 bacterial phyla. Across all recovery times, *Proteobacteria* emerged as the most abundant bacterial phylum, followed by *Acidobacteria*, *Actinobacteria*, and *Planctomycetes* (Figure 1). The non-metri multi-dimensional scaling (NMDS) analysis based on the Jaccard distance (number of OTUs) revealed substantial alterations in bacterial community composition over the recovery chronosequence (Figure 2). *Basidiomycota* abundance significantly increased and *Ascomycota* abundance decreased with recovery time, relative to R0. The fungal community structure significantly differed among R0, R5, R14, R16, and R19 (Figure 2).

### 2.4. Relationship between Soil Properties, Plant Communities, and Microbial Populations

**Figure 3:**
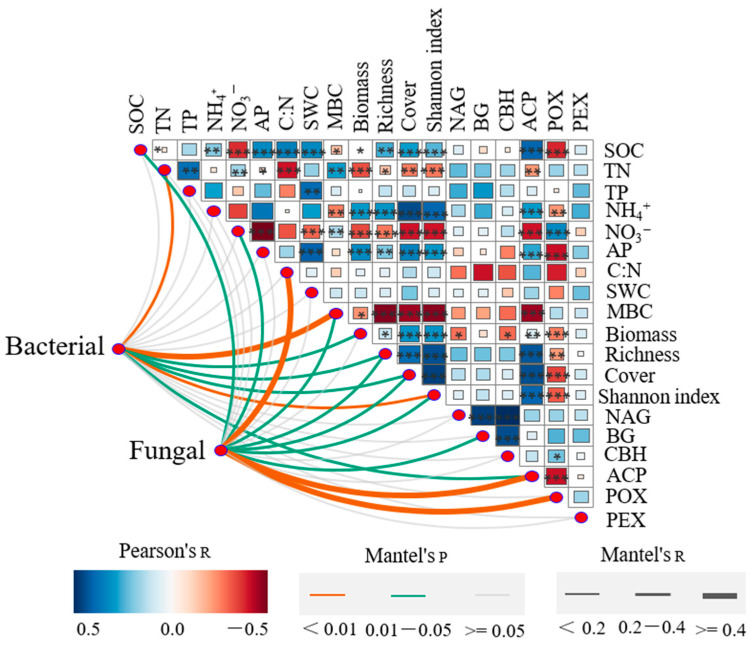
Correlation analysis between soil properties and plant communities, and the Mantel test for bacterial and fungal community composition (OTUs), soil properties, and plant communities. The colored lines indicate the significance of the Mantel test results: orange, *p* < 0.01; green, 0.01 < *p* < 0.05; gray, *p* ≥ 0.05. Line thickness indicates correlation coefficient size. Asterisks indicate the significance effect: *, *p* < 0.05; **, *p* < 0.01; ***, *p* < 0.001.

**Figure 4:**
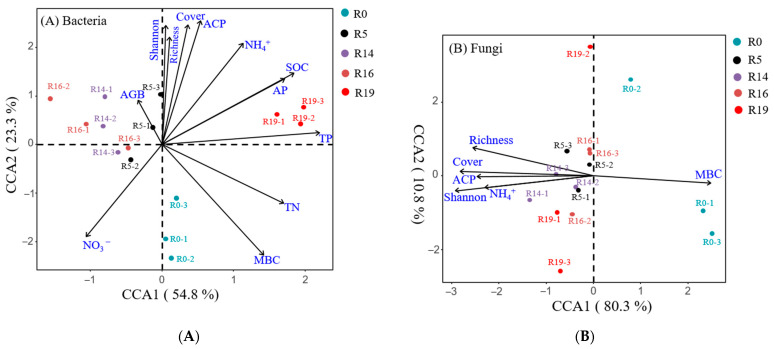
Canonical correspondence analysis (CCA) depicting the impact of soil and plant properties on bacterial (**A**) and fungal (**B**) communities across five treatments (R0, R5, R14, R16, and R19). R0: highly degraded grassland; R5: recovery time of 5 years; R14: recovery time of 14 years; R16: recovery time of 16 years; R19: recovery time of 19 years. The CCA analysis includes only significant data based on the Mantel test, excluding non-significant variables for both microbial groups.

The Mantel test revealed significant correlations between overall bacterial and fungal community composition and plant richness, cover, Shannon index, MBC content, and soil ACP activity (*p* < 0.05, Mantel’s R ≥ 0.2, Figure 3). In addition, soil TN content and plant aboveground biomass significantly correlated with bacterial community composition, while SOC content, NO_3_^−^-N content, C: N ratio, and soil POX activity significantly correlated with fungal composition (Figure 3). The canonical correspondence analysis (CCA) analysis further indicated that plant diversity, cover, ACP activity, and NO_3_^−^-N content in the restored grassland played crucial roles in shaping bacterial community changes, while plant diversity, cover, and ACP influenced the fungal community (Figure 4).

**Figure 5:**
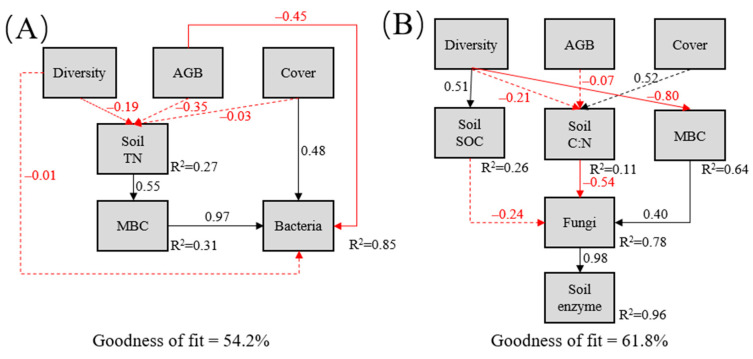
Partial least squares path models (PLS-PM) illustrating the relationships among plant communities, soil properties, and bacteria (**A**) and fungi (**B**). Red and black arrows indicate negative and positive causality flows, respectively. Numbers above lines indicate normalized path coefficients. Dotted red lines indicate non-significant path relationships. R^2^ values beside the latent variables denote coefficients of determination.

The partial least squares model (PLS-PM) indicated that the predictor variables collectively explained 85% and 78% of the variations in bacterial and fungal community composition, respectively (Figure 5). Plant coverage and soil TN positively affected bacterial community composition, whereas plant properties did not directly affect fungal community composition. Plant diversity and aboveground biomass negatively impacted fungal biomass and bacterial community composition, respectively. Moreover, the soil C: N ratio negatively affected fungal groups and positively affected soil enzymatic activities (Figure 5).

## 3. Discussion

### 3.1. Effect of Secondary Succession on Plant and Soil Properties

The observed secondary succession in the artificial grassland revealed a positive effect on plant communities, marked by increased species richness, diversity, and aboveground biomass. This outcome aligns with previous studies in different environments [17,22]. In contrast to dry environments, where initial increases in species richness and cover were followed by declines [23,24], our field sites in artificial plantations demonstrated an initial increase in plant cover and biomass. This finding suggests that rapid vegetation establishment through artificial planting benefits degraded alpine grassland restoration, particularly when natural seed propagation is limited.

The artificial cultivation of fine forage significantly improved soil nutrient levels compared to those in highly degraded grassland, due to the increased aboveground biomass and cover. The low nutrient contents at R0, such as SOC and TP, may be attributed to rapid plant litter loss due to high-intensity grazing, primarily by yaks. This grazing practice decreases aboveground biomass accumulation, increasing soil degradation [25]. The initial surge in vegetation and subsequent accumulation of aboveground biomass in the surface soil increased soil organic matter due to litter and root decomposition (Figure 3). In contrast to forested areas where aboveground plants supply abundant nutrients to the soil, our sites experienced lower aboveground biomass input due to winter grazing. The relatively constant SOC in R5 compared to R0 might be attributed to slow organic matter degradation under extreme climatic conditions (low temperatures and less oxygen) and intensive grazing (low biomass input). However, the stable soil C: N ratio over the chronosequence (Table 2) is consistent with previous reports indicating constant C: N ratios in soils [17,26,27]. The contribution of aboveground litter and belowground roots to soil nutrients, combined with the relatively constant plant diversity, might explain the stable C: N ratio in litter and roots [27,28]. This stability suggests that artificial grassland succession had a limited impact on soil C and nitrogen (N) levels in alpine degraded grassland. The similar trends in SOC, TN, TP, and NH_4_^+^-N contents and hydrolase (CBH, BG, ACP, and NAG) and oxidase (POX and PEX) enzyme activities align with previous findings that soil enzymatic activities are closely associated with soil nutrient contents [17,29]. The continuous increase in soil nutrients throughout the experiment indicates that 19 years was sufficient to observe clear patterns of secondary succession, but plant diversity and richness did not significantly differ between R5 and R19 (Table 1), most likely due to extreme climatic conditions [22].

### 3.2. Effect of Secondary Succession on Soil Microorganisms

Plant succession typically involves an initial increase in species richness, cover, and diversity, followed by a potential decrease due to strong competition or grazing pressure [22]. We found a clear succession pattern in soil microbial communities, characterized by changes in biomass, activities, and composition along the chronosequence. These findings align with previous studies by Zhang et al. [17] and Cheng et al. [25], reporting adverse effects of long-term grazing exclusion on soil bacterial diversity. Lozano et al. [22] reported an increase in bacterial diversity along a successional chronosequence of restored farmland, consistent with results reported for moorland [30] and agricultural systems [31]. However, Kuramae et al. [32] observed a significant overlap in microbial communities, with no clear differences between succession chronosequences in chalk grassland. These variations suggest that distinct successional patterns in microbial composition arise from different environmental conditions, such as drought, high altitude, and low soil nutrients.

Typically, changes in aboveground biomass lead to shifts in bacterial communities from fast-growing oligotrophic groups to slow-growing copiotrophic groups [33], which usually dominate communities with low resource availability [34]. In contrast, communities harboring *k* strategists tend to dominate soils with high resource availability [35]. The difference in soil substrates drives this shift, which is consistent with the increased SOC contents observed among the successional stages in our study. Despite this shift, the abundance of bacterial community groups did not significantly differ among the successional stages, indicating that a relatively short time (19 years) of plant recovery may not significantly alter bacterial community abundance in the artificial alpine grasslands.

Our results also revealed differences in fungal community composition with increasing recovery time (Figure 2), aligning with findings by Zhang et al. [17] and Banning et al. [35], who reported noticeable changes in fungal community composition during long-term grazing exclusion and succession from grassland to forest. However, Davey et al. [36] found no clear succession pattern in fungal groups under natural restoration in Arctic glacial sites. These contrasting results may be attributed to soil substrate availability along the chronosequence, with sufficient soil nutrients in our study, whereas the study by Davey et al. [36] began on glacial land with poor soil nutrients. Moreover, in our study, specific fungal groups, such as *Basidiomycota*, had increased relative abundance, indicating microbial succession in the soil. These changes in bacterial and fungal community structure suggest distinct successional patterns in microbial communities during the long-term restoration of degraded alpine grassland.

### 3.3. Relationships among Plants, Soil Properties, and Microbial Succession

Numerous studies have highlighted substantial differences in soil microbial (bacterial and fungal) community composition among vegetation succession stages [17,22,37]. Our results support this, demonstrating that plant and soil microbial community succession increased with recovery time, similar to findings by Zeng et al. [38] under long-term grazing exclusion and Lozano et al. [22] in a dry environment in the Tabernas Basin, Spain. These results highlight the significant impact of vegetation succession on soil microbial community structure. Increase plant cover with the chronosequence enhances soil organic matter and nutrient availability [22]. Over time, plant succession increases organic material input through coverage, biomass, and diversity changes. Our study found that plant cover and diversity (Shannon index) consistently and significantly affected bacterial and fungal community composition, microbial biomass, enzyme activity, and soil nutrient levels (Figure 3 and Figure 4). Similarly, Zhang et al. [17] reported that plant diversity directly affected bacterial and fungal communities. Conversely, Millard and Singh [39] found no correlation between bacterial and plant diversity. Increasing plant coverage with succession allows for better microclimatic conditions (such as temperature and soil moisture) in the topsoil layer [40]. Usually, increased plant cover enhances soil water-holding capacity, subsequently increasing soil moisture. However, our results showed that soil moisture content did not significantly correlate with plant cover (Figure 3). This finding contrasts with that of Zhang et al. [17], who found a close correlation between fungal communities and soil moisture in a semiarid environment. These results indicate that SMC substantially impacted soil microbial distribution in a semiarid region more than in a high-altitude area with sufficient precipitation. Moreover, soil organic matter characteristics are usually determined by the quantity and quality of the plant community, such as its biomass and diversity. Changes in soil microbial community result from changes in plant communities, ultimately determining soil nutrients (soil C and N). Thakur et al. [41] observed that higher plant diversity resulted in higher soil microbial community diversity, due to a more diverse organic matter composition from litter and root exudates. Therefore, plant diversity and cover can promote soil microbial growth and enhance microbial biomass due to their effects on SOC and microclimate conditions [42]. Our study supports this observation, with plant diversity (Shannon index) and cover associated with MBC, soil nutrients (SOC, TN), and enzyme activity (ACP and POX) (Figure 3 and Figure 5).

High plant biomass, cover, and diversity promoted by the increase in soil nutrients positively affected microbial communities in terms of biomass and activity, as reported for primary succession [43]. We found that TN content negatively correlated with plant biomass, cover, and diversity while positively driving the bacterial community (Figure 3 and Figure 5), consistent with results reported by Zhang et al. [17]. In our study, NO_3_^−^-N content only affected fungal groups, with no influence of NH_4_^+^-N content on bacterial and fungal groups (Figure 3). We also found that NO_3_^−^-N content decreased with successional time, while NH_4_^+^-N content increased, likely because many species prefer NO_3_^−^ as a nitrogen source [44]. These results indicate that only NO_3_^−^ forms of N contributed to plant and bacterial community succession. In addition, our results showed that phosphatase activity (such as ACP) increased with the chronosequence (Table 2) and its effects on bacterial and fungal community composition (Figure 3), which could be attributed to soil microorganisms sourcing scarce P from soil organic matter.

However, the PLS-PM methods used to characterize the relationship between the soil microbial community and environmental factors indicated that soil bacteria and fungi processed substrate decomposition differently (Figure 5). Plant diversity negatively correlated with fungal biomass, and the soil C: N ratio negatively drove the fungal community (Figure 3 and Figure 5). Given the increased plant diversity along the chronosequence and management model (winter pasture), most senescent plant straw did not contain enough available nutrients to meet soil microbial growth requirements [45]. Fungi can decompose recalcitrant organic matter such as lignin and cellulose better than bacteria [46]. However, soil organic matter needs enzymes like hydrolase and oxidase to degrade (Figure 5). Therefore, while some bacterial communities are actively involved in cellulose degradation [47], soil fungi seem to contribute strongly to the degradation of recalcitrant substrates. The predominance of soil fungi in the degradation of complex litter may be due to the ability of mycelium to explore soil space and acquire large reserves of soil nutrients [21], consistent with previous observations [46,48,49].

## 4. Materials and Methods

### 4.1. Study Sites

Our investigation focused on the degradation of swampy meadows within the Qinghai–Tibet Plateau, specifically in the Guoluo Tibetan Autonomous Prefecture of Qinghai Province, China. This region has undergone rehabilitation through external interventions, primarily artificial grassland. The study area (100°21′ E, 34°47′ N), characterized by a frigid alpine and semiarid climate, has been protected by artificial grassland to monitor the long-term restoration of *Heitutan*, as described by Gao and Li [5]. The experimental site has a mean annual precipitation of 350 mm, a mean annual temperature of about −4 °C, and an average elevation of 3700 m above sea level (a.s.l.). Additional details on environmental conditions, plant growth states, and local animal husbandry are available in Li et al. [4].

### 4.2. Experimental Design and Sampling

In July 2023, coinciding with peak aboveground productivity, we studied five grassland sites along a recovery chronosequence. Four of these sites, under artificial planting initially and subsequent natural restoration since 2004, 2007, 2009, and 2018, represented recovery periods of 19 years (R19), 16 years (R16), 14 years (R14), and 5 years (R5), respectively. *Elymus nutans* Griseb., *Poa pratensis* L., and *Festuca sinensis* Keng ex S.L.Lu were the three species used to restore the grasslands. The fifth site, denoted R0, served as a reference and featured highly degraded grassland covered with poisonous grass. All sites had a history of intensive winter grazing. The selected sites shared similar altitudes, slope aspects, slope gradients, soil types, and environmental conditions. We established three 20 m × 20 m plots within each site separated by 30 m. Soil samples were collected from the topsoil (0–20 cm) using a 4 cm diameter auger inserted at five randomly selected positions after removing the litter layer. The collected soil cores were combined into composite samples and stored in a low-temperature incubator. Stones, litter, roots, and animal bodies were removed, with the DNA extraction sample immediately stored at −80 °C. Additionally, three 50 cm × 50 cm subplots were randomly designated within each plot to estimate vegetation aboveground biomass, coverage, and species count (see Table S1). Aboveground biomass was determined after oven-drying at 60 °C.

### 4.3. Soil Chemical Analysis

Various soil properties were analyzed to understand changes over time. SOC content was determined using the Walkley and Black dichromate oxidation method with a correction factor of 1.08. Total nitrogen (TN) content was determined using the K_2_SO_4_–CuSO_4_–Se (100: 10: 1) distillation method. After perchloric acid digestion and ascorbic acid reduction, TP content was determined using the molybdate colorimetric method. Inorganic nitrogen (NH_4_^+^-N and NO_3_^−^-N) contents were analyzed using an auto-flow injection system (Auto-Analyzer AA3, Germany). The soil’s AP was quantified using the Olsen-P method [50]. SMC was determined after drying at 105 °C to constant weight. Soil pH was measured in a 1: 2.5 soil–water mixture using a pH meter (Seven Excellence S400).

### 4.4. Microbial Biomass and Enzymatic Activities

Soil MBC and MBN contents were determined by quantifying differences in carbon and nitrogen between chloroform-fumigated and non-fumigated soil samples. For soil enzyme assays, the activities of four hydrolytic enzymes involved in C, N, and P cycling (BG, CBH, NAG, and ACP) were measured [51]. We also assessed the activities of two oxidases (POX and PEX) that are responsible for degrading recalcitrant organic C [52]. Hydrolytic enzyme activities were measured using fluorescence microplate assay with 4-methylumbelliferone (MUB)-labeled substrate [53]. Oxidases were measured spectrophotometrically in a clear 96-well microplate using the substrate L-3,4-dihydroxyphenylalanine (DOPA) [29]. Activity levels were determined fluorometrically with a plate reader (Varioskan LUX, ThermoFisher Scientific, Waltham, MA, USA) set at 450 nm emission and 365 nm excitation, with the results expressed in units of substrate converted (μmol) per mL of sample (μmol g^−1^ h^−1^).

### 4.5. Soil DNA Extraction and Sequencing

Microbial deoxyribonucleic acid (DNA) extraction was performed on each soil sample using a QIAamp^®^ DNA Stool Mini Kit for soil (Qiagen, Hilden, Germany) following the manufacturer’s instructions, with the DNA concentration and purity assessed using a Nanodrop ND-1000. Bacterial genomic DNA was used to amplify the V3–V4 hypervariable region of the 16SrRNA gene, using the primers 515F: GTGCCAGCMGCCGCGG and 907R: CCGTCAATTCMTTTRAGTTT. The fungal ITS2 region was amplified using primers ITS3: GCATCGATGAAGAACGCAGC and ITS4: TCCTCCGCTTATTGATATGC, under the same conditions. Each sample underwent independent amplifications in triplicate. The polymerase chain reaction (PCR) products were checked by agarose gel electrophoresis, and then pooled to use as a template. Index PCR was performed using index primers to add the Illumina index to the library. Amplification products were checked using gel electrophoresis and purified using an Agencourt AMPure XP Kit (Beckman Coulter, Brea, CA, USA). The purified products were indexed in the 16S V3–V4 library. The library quality was assessed using a Qubit@2.0 Fluorometer (ThermoFisher Scientific) and Agilent Bioanalyzer 2100 systems (Agilent, Santa Clara, CA, USA). All sequence data have been deposited in the CNGB Sequence Archive (CNSA) of the China National GeneBank DataBase (CNGBdb) (accession number CNP0005274).

### 4.6. Bioinformatics Analysis

Paired-end clean reads were merged using fast length adjustment of short reads (FLASH, v1.2.11) and quality-filtered, based on prescribed conditions, to obtain high-quality clean reads that were clustered into OTUs using UPARSE with 97% similarity. All OTUs were classified based on the Ribosomal Database Project by Mothur. Alpha diversities, including the Shannon–Wiener index, Chao1 estimator, and ACE, were analyzed for bacterial and fungal communities by Mothur. NMDS analysis based on OTUs was performed using R Project (Vegan package, V3.3.1).

### 4.7. Statistical Analysis

The effect of recovery length on plant properties (including aboveground biomass, coverage, and diversity), soil properties, microbial biomass, enzyme activities, and microbial diversity was assessed using one-way ANOVA, followed by Tukey’s honestly significant difference test for pairwise comparisons. Plant community diversity was assessed using the Shannon–Wiener index. NMDS analysis based on OTU levels evaluated the relationships between bacterial and fungal community structures during succession stages. The Mantel test was performed using the ‘dplyr’ R package (Vegen package, V3.3.1) to assess correlations between plant characteristics, soil properties, and soil microbial community composition. (CCA was conducted using the ‘vegan’ R package to assess environmental factors influencing bacterial and fungal community structure. A PLS-PM was performed using the ‘plspm’ R package to infer potential direct and indirect effects of diversity, aboveground biomass, plant cover, soil C, N, and C: N ratio, and MBC on bacterial and fungal community composition. Significance was set at *p* ˂ 0.05. One-way ANOVAs and multiple comparisons were conducted using Genstat 18 software (VSN International Ltd., Rothamsted, UK), with figures generated using Origin 9.2 software (OriginLab 2016, Northampton, MA, USA).

## 5. Conclusions

Our study demonstrates the significant recovery of degraded alpine grassland through above- and belowground succession processes. Notable changes in plant biomass, cover, and diversity, soil properties, soil microbial biomass, diversity, and community structure characterize this restoration. Rapid establishment of plant coverage on heavily degraded grassland could benefit restoration efforts, suggesting the efficacy of strategies like alternate-year grazing in alpine grasslands on the Qinghai–Tibet Plateau. The parallel occurrence of plant and fungal succession highlights the interconnectedness of above- and belowground processes. Bacterial succession was influenced by plant biomass, cover, and soil TN content, while fungal succession was controlled by plant diversity and the soil C: N ratio. Changes in fungal communities were also susceptible to variations in soil enzyme activity, including phosphatase. Our findings underscore the importance of plant biomass, cover, and diversity in shaping soil microbial community composition. These factors likely influence soil nutrient dynamics and microclimate conditions. This study enhances our understanding of grassland ecosystem functioning and offers valuable insights for managing alpine grassland recovery.

## Figures and Tables

**Table 1:** Plant characteristics with secondary succession time. Values are mean ± SE (n = 3). *F* and *p* values indicate the significance of one-way analysis of variance (ANOVA). Different letters within a row indicate significant differences between treatments (*p* < 0.05).

Parameters	R0	R5	R14	R16	R19	*F*	*p*
Aboveground biomass (kg ha^−1^)	1098 ± 74.8 ^c^	1476 ± 29.8 ^b^	2253 ± 52.8 ^a^	1016 ± 16.1 ^c^	1437 ± 76.9 ^d^	77.57	<0.001
Cover (%)	67.7 ± 2.60 ^c^	90.7 ± 2.60 ^ab^	96.3 ± 2.03 ^a^	87.0 ± 1.53 ^b^	96.3 ± 2.33 ^a^	27.49	<0.001
Richness	10.3 ± 1.33 ^b^	19.7 ± 1.20 ^a^	18.3 ± 1.33 ^a^	20.0 ± 1.15 ^a^	20.3 ± 1.20 ^a^	11.37	0.001
Shannon Index	2.06 ± 0.05 ^c^	2.61 ± 0.05 ^ab^	2.77 ± 0.05 ^a^	2.55 ± 0.07 ^b^	2.69 ± 0.09 ^ab^	18.02	0.0001

R0: highly degraded grassland; R5: recovery time of 5 years; R14: recovery time of 14 years; R16: recovery time of 16 years; R19: recovery time of 19 years.

**Table 2:** Soil properties with secondary succession time. Values are mean ± SE (n = 3). *F* and *p* values indicate the significance of one-way ANOVA. Different letters within a row indicate significant differences between treatments (*p* < 0.05).

Parameters	R0	R5	R14	R16	R19	*F*	*p*
SOC (g kg^−1^)	32.4 ± 0.28 ^c^	32.8 ± 0.40 ^c^	35.2 ± 2.26 ^a^	36.8 ± 0.29 ^b^	42.6 ± 0.38 ^a^	15.0	<0.001
TN (g kg^−1^)	3.12 ± 0.08 ^b^	3.65 ± 0.18 ^a^	3.05 ± 0.07 ^b^	3.22 ± 0.08 ^b^	3.68 ± 0.05 ^a^	8.93	0.002
TP (g kg^−1^)	0.40 ± 0.01 ^b^	0.43 ± 0.01 ^a^	0.39 ± 0.01 ^b^	0.38 ± 0.00 ^b^	0.44 ± 0.00 ^a^	10.4	0.001
NO_3_^−^-N (mg kg^−1^)	6.65 ± 1.11 ^a^	5.94 ± 0.06 ^a^	3.62 ± 0.11 ^b^	5.61 ± 0.02 ^a^	3.42 ± 0.38 ^b^	7.46	0.005
NH_4_^+^-N (mg kg^−1^)	5.98 ± 0.47 ^b^	9.89 ± 0.62 ^a^	9.31 ± 0.06 ^a^	7.02 ± 0.49 ^b^	10.2 ± 0.19 ^a^	19.9	<0.001
AP (mg kg^−1^)	2.65 ± 0.07 ^cd^	2.94 ± 0.03 ^c^	3.83 ± 0.07 ^b^	2.56 ± 0.18 ^d^	4.24 ± 0.14 ^a^	43.8	<0.001
pH	8.05 ± 0.01 ^a^	7.72 ± 0.03 ^b^	6.65 ± 0.09 ^c^	7.99 ± 0.05 ^a^	7.86 ± 0.12 ^ab^	68.6	<0.001
C:N ratio	10.4 ± 0.27 ^ab^	9.07 ± 0.43 ^b^	11.6 ± 0.89 ^a^	11.5 ± 0.37 ^a^	11.6 ± 0.14 ^a^	4.92	0.019
MBC (mg kg^−1^)	744 ± 12.7 ^a^	468 ± 16.2 ^c^	383 ± 5.02 ^e^	430 ± 0.52 ^d^	552 ± 5.90 ^b^	208	<0.001
MBN (mg kg^−1^)	85.1 ± 3.64 ^a^	72.5 ± 0.96 ^b^	68.2 ± 1.32 ^b^	67.0 ± 0.48 ^b^	70.8 ± 3.31 ^b^	9.65	0.002
MBP (mg kg^−1^)	8.63 ± 1.85 ^d^	30.2 ± 2.33 ^b^	39.5 ± 0.84 ^a^	18.9 ± 0.78 ^c^	8.85 ± 1.88 ^d^	66.6	<0.001
SMC (%)	25.0 ± 0.55 ^b^	25.7 ± 0.64 ^b^	25.7 ± 0.24 ^b^	22.0 ± 0.98 ^c^	32.7 ± 1.42 ^a^	20.6	<0.001
NAG (μmol g^−1^ h^−1^)	60.7 ± 3.87 ^c^	210 ± 23.6 ^a^	145 ± 5.88 ^b^	32.5 ± 9.88 ^c^	146 ± 17.2 ^b^	26.0	<0.001
BG (μmol g^−1^ h^−1^)	184 ± 0.79 ^d^	1010 ± 62.0 ^a^	364 ± 17.5 ^c^	197 ± 24.1 ^d^	479 ± 6.16 ^b^	120	<0.001
CBH (μmol g^−1^ h^−1^)	83.2 ± 2.15 ^d^	163 ± 10.4 ^a^	138 ± 10.50 ^b^	71.5 ± 1.10 ^d^	108 ± 4.23 ^c^	29.6	<0.001
ACP (μmol g^−1^ h^−1^)	412 ± 2.05 ^d^	630 ± 28.8 ^c^	695 ± 15.6 ^b^	779 ± 15.2 ^a^	830 ± 1.94 ^a^	101	<0.001
POX (nmol g^−1^ h^−1^)	262 ± 11.1 ^a^	280 ± 0.94 ^a^	224 ± 9.13 ^b^	206 ± 11.5 ^b^	199 ± 7.57 ^b^	15.6	<0.001
PEX (nmol g^−1^ h^−1^)	85.0 ± 1.40 ^ab^	96.0 ± 1.87 ^a^	70.6 ± 5.39 ^b^	83.8 ± 9.40 ^ab^	92.5 ± 1.70 ^a^	3.81	0.039

R0: highly degraded grassland; R5: recovery time of 5 years; R14: recovery time of 14 years; R16: recovery time of 16 years; R19: recovery time of 19 years. MBP: microbial biomass phosphorus; SMC: soil moisture content; PEX: peroxidase.

**Table 3:** Soil microbial diversities with secondary succession time. Values are mean ± SE (n = 3). *F* and *p* values indicate the significance of one-way ANOVA. Different letters within a row indicate significant differences between treatments (*p* < 0.05).

Parameters		R0	R5	R14	R16	R19	*F*	*p*
Observed OTUs	Bacteria	2822 ± 9.23 ^a^	2622 ± 49.0 ^b^	2533 ± 16.6 ^b^	2598 ± 31.1 ^b^	2754 ± 38.8 ^a^	14.1	0.0004
	Fungi	837 ± 14.4 ^a^	850 ± 19.6 ^a^	679 ± 9.50 ^b^	672 ± 21.9 ^b^	673 ± 9.50 ^b^	34.3	<0.0001
Chao1	Bacteria	2902 ± 8.13 ^a^	2715 ± 14.2 ^c^	2543 ± 4.79 ^e^	2685 ± 1.38 ^d^	2764 ± 8.18 ^b^	236	<0.0001
	Fungi	838 ± 14.0 ^a^	851 ± 19.5 ^a^	679 ± 9.90 ^b^	673 ± 21.8 ^b^	674 ± 9.70 ^b^	34.7	<0.0001
ACE	Bacteria	2895 ± 10.9 ^a^	2712 ± 12.2 ^c^	2541 ± 4.68 ^e^	2673 ± 1.99 ^d^	2762 ± 7.12 ^b^	242	<0.0001
	Fungi	839 ± 14.1 ^a^	852 ± 19.6 ^a^	680 ± 9.80 ^b^	675 ± 21.9 ^b^	675 ± 9.70 ^b^	34.1	<0.0001
Shannon index	Bacteria	7.29 ± 0.006 ^ab^	7.29 ± 0.021 ^a^	7.21 ± 0.006 ^c^	7.24 ± 0.021 ^bc^	7.30 ± 0.015 ^a^	6.73	0.0068
	Fungi	5.09 ± 0.17 ^ab^	5.33 ± 0.11 ^a^	4.85 ± 0.11 ^b^	4.75 ± 0.14 ^bc^	4.41 ± 0.05 ^c^	8.37	0.0031

R0: highly degraded grassland; R5: recovery time of 5 years; R14: recovery time of 14 years; R16: recovery time of 16 years; R19: recovery time of 19 years.

## Data Availability

The data presented in this study and Supplementary Materials are available on request from the corresponding author.

## Supplementary Materials

The following supporting information can be downloaded at: https://www.mdpi.com/article/10.3390/plants13060780/s1, Table S1 Plant species along the different secondary succession time. ‘+’ symbols indicates the plant species exist in a given succession time.

## References

[1] Gao J., Li X.L. (2016). Degradation of frigid swampy meadows on the Qinghai–Tibet Plateau. Prog. Phys. Geogr..

[2] Li X.L., Perry G.L.W., Brierley G., Sun H.Q., Li C.H., Lu G.X. (2012). Quantitative assessment of degradation classifications for degraded alpine meadows (heitutan), Sanjiangyuan, Western China. Land Degrad. Dev..

[3] Li X.L., Xue Z.P., Gao J. (2016). Dynamic changes of plateau wetlands in Madou county, the Yellow River source zone of China: 1990–2013. Wetlands.

[4] Li X.L., Xue Z.P., Gao J. (2016). Environmental influence on vegetation properties of frigid wetlands on the Qinghai-Tibet Plateau, Western China. Wetlands.

[5] Gao J., Li X.L. (2017). A knowledge-based approach to mapping degraded meadows on the Qinghai–Tibet Plateau, China. Int. J. Remote Sens..

[6] Fierer N., Wood S.A., Bueno de Mesquita C.P. (2021). How microbes can, and cannot, be used to assess soil health. Soil Biol. Biochem..

[7] Coban O., De Deyn G.B., van der Ploeg M. (2022). Soil microbiota as game-changers in restoration of degraded lands. Science.

[8] Van der Putten W.H., Bardgett R.D., Bever J.D., Bezemer T.M., Casper B.B., Fukami T., Kardol P., Klironomos J.N., Kulmatiski A., Schweitzer J.A. (2013). Plant–soil feedbacks: The past, the present and future challenges. J. Ecol..

[9] Cui Y.X., Fang L.C., Guo X.B., Wang X., Zhang Y.J., Li P.F., Zhang X.C. (2018). Ecoenzymatic stoichiometry and microbial nutrient limitation in rhizosphere soil in the arid area of the northern Loess Plateau, China. Soil Biol. Biochem..

[10] Cui Y.X., Wang X., Zhang X.C., Ju W.L., Duan C.J., Guo X.B., Wang Y.Q., Fang L.C. (2020). Soil moisture mediates microbial carbon and phosphorus metabolism during vegetation succession in a semiarid region. Soil Biol. Biochem..

[11] Haichar F.e.Z., Marol C., Berge O., Rangel-Castro J.I., Prosser J.I., Balesdent J., Heulin T., Achouak W. (2008). Plant host habitat and root exudates shape soil bacterial community structure. ISME J..

[12] Kardol P., De Deyn G.B., Laliberté E., Mariotte P., Hawkes C.V., van der Putten W. (2013). Biotic plant–soil feedbacks across temporal scales. J. Ecol..

[13] Bitas V., Kim H.-S., Bennett J.W., Kang S. (2013). Sniffing on microbes: Diverse roles of microbial volatile organic compounds in plant health. Mol. Plant. Microbe Interact..

[14] Wang J., Liu G.B., Zhang C., Wang G.L., Fang L.C., Cui Y.X. (2019). Higher temporal turnover of soil fungi than bacteria during long-term secondary succession in a semiarid abandoned farmland. Soil Till. Res..

[15] Zhong Y.Q.W., Yan W.M., Wang R.W., Wang W., Shangguan Z.P. (2018). Decreased occurrence of carbon cycle functions in microbial communities along with long-term secondary succession. Soil Biol. Biochem..

[16] Sun S., Li S., Avera B.N., Strahm B.D., Badgley B.D., Löffler F.E. (2017). Soil bacterial and fungal communities show distinct recovery patterns during forest ecosystem restoration. Appl. Environ. Microbiol..

[17] Zhang C., Liu G.B., Song Z., Wang J., Guo L. (2018). Interactions of soil bacteria and fungi with plants during long-term grazing exclusion in semiarid grasslands. Soil Biol. Biochem..

[18] Schmidt S.K., Nemergut D.R., Darcy J.L., Lynch R. (2014). Do bacterial and fungal communities assemble differently during primary succession. Mol. Ecol..

[19] Rousk J., Bååth E. (2007). Fungal biomass production and turnover in soil estimated using the acetate-in-ergosterol technique. Soil Biol. Biochem..

[20] Cui Y.X., Fang L.C., Guo X.B., Han F., Ju W.L., Ye L.P., Wang X., Tan W.F., Zhang X.C. (2019). Natural grassland as the optimal pattern of vegetation restoration in arid and semi-arid regions: Evidence from nutrient limitation of soil microbes. Sci. Total Environ..

[21] Panchal P., Preece C., Peñuelas J., Giri J. (2022). Soil carbon sequestration by root exudates. Trends Plant Sci..

[22] Lozano Y.M., Hortal S., Armas C., Pugnaire F.I. (2014). Interactions among soil, plants, and microorganisms drive secondary succession in a dry environment. Soil Biol. Biochem..

[23] Scott A.J., Morgan J.W. (2012). Recovery of soil and vegetation in semi-arid Australian old fields. J. Arid Environ..

[24] Dana E.D., Mota J.F. (2006). Vegetation and soil recovery on gypsum outcrops in semi-arid Spain. J. Arid Environ..

[25] Cheng J.M., Jing G.H., Wei L., Jing Z.B. (2016). Long-term grazing exclusion effects on vegetation characteristics, soil properties and bacterial communities in the semi-arid grasslands of China. Ecol. Eng..

[26] Baddeley J.A., Edwards A.C., Watson C.A. (2017). Changes in soil C and N stocks and C:N stoichiometry 21 years after land use change on an arable mineral topsoil. Geoderma.

[27] Zeng Q.C., Liu Y., Fang Y., Ma R.T., Lal R., An S.S., Huang Y.M. (2017). Impact of vegetation restoration on plants and soil C:N:P stoichiometry on the Yunwu Mountain Reserve of China. Ecol. Eng..

[28] Yang Y.h., Luo Y.Q. (2011). Carbon: Nitrogen stoichiometry in forest ecosystems during stand development. Glob. Ecol. Biogeogr..

[29] Han C.L., Zhou W.D., Gu Y.J., Wang J.Q., Zhou Y.F., Xue Y.Y., Shi Z.G., Siddique K.H.M. (2024). Effects of tillage regime on soil aggregate-associated carbon, enzyme activity, and microbial community structure in a semiarid agroecosystem. Plant Soil.

[30] Mitchell R.J., Hester A.J., Campbell C.D., Chapman S.J., Cameron C.M., Hewison R.L., Potts J.M. (2011). Explaining the variation in the soil microbial community: Do vegetation composition and soil chemistry explain the same or different parts of the microbial variation?. Plant Soil.

[31] Jangid K., Williams M.A., Franzluebbers A.J., Schmidt T.M., Coleman D.C., Whitman W.B. (2011). Land-use history has a stronger impact on soil microbial community composition than aboveground vegetation and soil properties. Soil Biol. Biochem..

[32] Kuramae E., Gamper H., van Veen J., Kowalchuk G. (2011). Soil and plant factors driving the community of soil-borne microorganisms across chronosequences of secondary succession of chalk grasslands with a neutral pH. FEMS Microbiol. Ecol..

[33] Zhou Z., Wang C., Jiang L., Luo Y. (2017). Trends in soil microbial communities during secondary succession. Soil Biol. Biochem..

[34] Fierer N., Bradford M.A., Jackson R.B. (2007). Toward an ecological classification of soil bacteria. Ecology.

[35] Banning N.C., Gleeson D.B., Grigg A.H., Grant C.D., Andersen G.L., Brodie E.L., Murphy D.V. (2011). Soil microbial community successional patterns during forest ecosystem restoration. Appl. Environ. Microbiol..

[36] Davey M., Blaalid R., Vik U., Carlsen T., Kauserud H., Eidesen P.B. (2015). Primary succession of *Bistorta vivipara* (L.) Delabre (Polygonaceae) root-associated fungi mirrors plant succession in two glacial chronosequences. Environ. Microbiol..

[37] Fry E.L., Manning P., Macdonald C., Hasegawa S., De Palma A., Power S.A., Singh B.K. (2016). Shifts in microbial communities do not explain the response of grassland ecosystem function to plant functional composition and rainfall change. Soil Biol. Biochem..

[38] Zeng Q.C., An S.S., Liu Y. (2017). Soil bacterial community response to vegetation succession after fencing in the grassland of China. Sci. Total Environ..

[39] Millard P., Singh B.K. (2010). Does grassland vegetation drive soil microbial diversity?. Nutr. Cycl. Agroecosyst..

[40] Pugnaire F.I., Armas C., Valladares F. (2004). Soil as a mediator in plant-plant interactions in a semi-arid community. J. Veg. Sci..

[41] Thakur M.P., Milcu A., Manning P., Niklaus P.A., Roscher C., Power S., Reich P.B., Scheu S., Tilman D., Ai F. (2015). Plant diversity drives soil microbial biomass carbon in grasslands irrespective of global environmental change factors. Glob. Chang. Biol..

[42] Steinauer K., Tilman D., Wragg P.D., Cesarz S., Cowles J.M., Pritsch K., Reich P.B., Weisser W.W., Eisenhauer N. (2015). Plant diversity effects on soil microbial functions and enzymes are stronger than warming in a grassland experiment. Ecology.

[43] Tscherko D., Hammesfahr U., Zeltner G., Kandeler E., Böcker R. (2005). Plant succession and rhizosphere microbial communities in a recently deglaciated alpine terrain. Basic Appl. Ecol..

[44] Boudsocq S., Niboyet A., Lata J.C., Raynaud X., Loeuille N., Mathieu J., Blouin M., Abbadie L., Barot S. (2012). Plant preference for ammonium versus nitrate: A neglected determinant of ecosystem functioning?. Am. Nat..

[45] Fontaine S., Henault C., Aamor A., Bdioui N., Bloor J.M.G., Maire V., Mary B., Revaillot S., Maron P.A. (2011). Fungi mediate long term sequestration of carbon and nitrogen in soil through their priming effect. Soil Biol. Biochem..

[46] Santiago T., Pablo L.P., Olga S.C., Veronica G., Marina G.-P. (2021). Soil microbial communities respond to an environmental gradient of grazing intensity in south Patagonia Argentina. J. Arid Environ..

[47] El Zahar Haichar F., Achouak W., Christen R., Heulin T., Marol C., Marais M.F., Mougel C., Ranjard L., Balesdent J., Berge O. (2007). Identification of cellulolytic bacteria in soil by stable isotope probing. Environ. Microbiol..

[48] Poll C., Marhan S., Ingwersen J., Kandeler E. (2008). Dynamics of litter carbon turnover and microbial abundance in a rye detritusphere. Soil Biol. Biochem..

[49] Shi J.Y., Gong J.R., Li X.B., Zhang Z.H., Zhang W.Y., Li Y., Song L.Y., Zhang S.Q., Dong J., Baoyin T.-t. (2022). Plant–microbial linkages regulate soil organic carbon dynamics under phosphorus application in a typical temperate grassland in northern China. Agric. Ecosyst. Environ..

[50] Yuan Z.Q., Yu K.L., Epstein H., Fang C., Li J.T., Liu Q.Q., Liu X.W., Gao W.J., Li F.M. (2016). Effects of legume species introduction on vegetation and soil nutrient development on abandoned croplands in a semi-arid environment on the Loess Plateau, China. Sci. Total Environ..

[51] Sinsabaugh R.L., Hill B.H., Follstad Shah J.J. (2009). Ecoenzymatic stoichiometry of microbial organic nutrient acquisition in soil and sediment. Nature.

[52] Sinsabaugh R.L. (2010). Phenol oxidase, peroxidase and organic matter dynamics of soil. Soil Biol. Biochem..

[53] Bell C.W., Fricks B.E., Rocca J.D., Steinweg J.M., McMahon S.K., Wallenstein M.D. (2013). High-throughput fluorometric measurement of potential soil extracellular enzyme activities. JOVE J. Vis. Exp..

