# Supplementary material for: Soil, Plant, and Microorganism Interactions Drive Secondary Succession in Alpine Grassland Restoration
Source: Plants (Basel). 2024 Mar 9;13(6):780. doi: 10.3390/plants13060780 (PMC10975169; doi:10.3390/plants13060780)
Supplement: Supplementary file 1 [file plants-13-00780-s001.zip › plants-2875368-supplementary.pdf]

Table S1 Plant species along the different secondary succession time. '+'symbols indicates the plant species exist in a given succession time.

Species	R0	R5	R14	R16	R19
Ligularia virgaurea (Maxim.) Mattf.			+	+	+
Oxytropis ochrocephala Bunge		+	+	+	+
Gentiana straminea Maxim.		+	+	+	+
Kobresia humilis (C. A. Mey ex Trauvt.) Sergievskaya.		+	+	+	+
Poa crymophila Keng ex C.Ling		+	+	+	+
Poa pratensis L.		+	+	+	+
Elymus nutans Griseb.		+	+	+	+
Koeleria litvinowii Domin		+	+	+	+
Tibetia himalaica (Baker) H. P. Tsui		+	+	+	+
Lancea tibetica Hook.f. et Thoms.	+	+	+	+	+
Astragalus polycladus Bur. et Franch.		+	+	+	+
Melandrium apricum (Turcz.) Rohrb.		+	+	+	+
Aconitum flavam Hand-Mazz.		+	+		+
Scirpus pumilus Vahl					+
Halenia elliptica D. Don		+	+	+	+
Ranunculus tanguticus (Maxim.) Ovcz.		+		+	+
Ranunculus tanguticus (Maxim.) Ovcz. var. capillaceus (Franch.) L.Liou		+	+		+
Taraxacum mongolicum Hand.-Mazz.			+	+	+
Potentilla anserina Linn.	+	+			+
Potentilla multifolia L.		+	+	+	+
Plantago depressa Willd.		+		+	+
Euphrasia pectinata Ten.					+
Hypecoum leptocarpum Hook.	+				+
Pedicularis kansuensis Maxim.		+	+	+	+
Artemisia hedinii Ostenf. et Pauls.					+
Festuca parrigluma Steud			+		+
Medicago ruthenica (L.) Trautv.			+		+
Anaphalis lactea Maxim.		+	+		+
Stellera chamaejasme L.				+	
Carex atrofusca Schkuhr subsp. *minor* (Boott) T. Koyama	+		+	+	
Ajania tenuifolia (Jacq.) Tzvel.	+	+		+	
Gentiana abaensis T.N.Ho.	+	+	+	+	
Potentilla saundersiana Rogle				+	
Kobresia pygmaea (C. B. Clarke) C. B. Clarke			+	+	
Euphorbia fischeriana auct. non Steud.		+		+	
Veronica eriogyne H. Winkl.	+		+	+	
Festuca sinensis Keng ex S.L.Lu			+	+	
Euphorbia altotibetica O. Pauls.				+	
Leontopodium nanum (Hook. f. et Thoms) Hand.-Mazz.		+	+	+	
Saussurea stella Maxim.				+	
Thalictrum rutifolium Hook.f. et Thoms			+		

Ajuga lupulina Maxim.		+	+
Aster alpinus Linn.		+	+
Carum buriaticum Turcz.f.			+
Stipa aliena Keng			+
Sanguisorba filiformis (Hook. f.) Hand.-Mazz.		+	+
Artemisia sieversiana Ehrhart ex Willd.		+	
Cirsium souliei (Franch.) Mattf.	+		
Elsholtzia densa Benth.	+		
Polygonum sibiricum L.	+		
Achnatherum inebrians (Hance) Keng ex Tzvel.	+		
Chenopodium glaucum L.	+		
Descurainia Sophia (Linn.) Schur.	+		
Glaux mantima L.	+		
Delphinium kamaonense Hunth var. glabrescens W.T.Wang	+		
Thlaspi arvense Linn.	+		
